# Mechanisms of IS*26*-Mediated Amplification of the *aphA1* Gene Leading to Tobramycin Resistance in an Acinetobacter baumannii Isolate

**DOI:** 10.1128/spectrum.02287-22

**Published:** 2022-09-08

**Authors:** Christopher J. Harmer, Francois Lebreton, Jason Stam, Patrick T. McGann, Ruth M. Hall

**Affiliations:** a School of Life and Environmental Sciences, The University of Sydney, Sydney, New South Wales, Australia; b Multidrug Resistant Organism Repository and Surveillance Network, Walter Reed Army Institute of Research, Silver Spring, Maryland, USA; University at Albany, State University of New York

**Keywords:** IS*26*, antibiotic resistance, gene amplification, heteroresistance, tobramycin resistance

## Abstract

Enhanced levels of resistance to antibiotics arising from amplification of an antibiotic resistance gene that impact therapeutic options are increasingly observed. Amplification can also disclose novel phenotypes leading to treatment failure. However, the mechanism is poorly understood. Here, the route to amplification of the *aphA1* kanamycin and neomycin resistance gene during tobramycin treatment of an Acinetobacter baumannii clinical isolate, leading to tobramycin resistance and treatment failure, was investigated. In the tobramycin-susceptible parent isolate, MRSN56, a single copy of *aphA1* is present in the pseudocompound transposon PTn*6020*, bounded by directly oriented copies of IS*26*. For two clinical resistant isolates, new long-read sequence data were combined with available short-read data to complete the genomes. Comparison to the completed genome of MRSN56 revealed that, in both cases, IS*26* had generated a circular translocatable unit (TU) containing PTn*6020* and additional adjacent DNA. In one case, this TU was reincorporated into the second product generated by the deletion that formed the TU via the targeted conservative route and amplified about 7 times. In the second case, the TU was incorporated at a new location via the copy-in route and amplified about 65 times. Experimental amplification *ex vivo* by subjecting MRSN56 to tobramycin selection pressure yielded different TUs, which were incorporated at either the original location or a new location and amplified many times. The outcomes suggest that when IS*26* is involved, amplification occurs via rolling circle replication of a newly formed TU coupled to the IS*26*-mediated TU formation or reincorporation step.

**IMPORTANCE** Heteroresistance, a significant issue that is known to impact antibiotic treatment outcomes, is caused by the presence of spontaneously arising cells with elevated levels of resistance to therapeutically important antibiotics in a population of susceptible cells. Gene amplification is one well-documented cause of heteroresistance, but precisely how extensive amplification occurs is not understood. Here, we establish the case for the direct involvement of IS*26* activity in the amplification of the *aphA1* gene to disclose resistance to tobramycin. The *aphA1* gene is usually found associated with IS*26* in Gram-negative pathogens and is commonly found in extensively resistant Acinetobacter baumannii strains. IS*26* and related IS cause adjacent deletions, forming a nonreplicating circular molecule known as a translocatable unit (TU), and amplification via a rolling circle mechanism appears to be coupled to either IS*26*-mediated TU formation or reincorporation. Related IS found in Gram-positive pathogens may play a similar role.

## INTRODUCTION

Heteroresistance caused by an antibiotic-resistant subpopulation in a culture of a susceptible bacterial strains is receiving increasing attention as a source of antibiotic treatment failure (1). Gene duplication or further amplification is a known cause of heteroresistance, but the mechanism is poorly understood (2, 3). Unequal crossing over between directly oriented repeats in copies of the chromosome present in the same cell can lead to duplication of a gene or region surrounded by the directly oriented identical DNA segments, for example, IS elements. Then, this process can be repeated using the longer duplication generated to increase the number of gene copies. However, this proposed mechanism may not be able to readily explain recent reports of amplification to very high levels (4–6).

**FIG 1:**
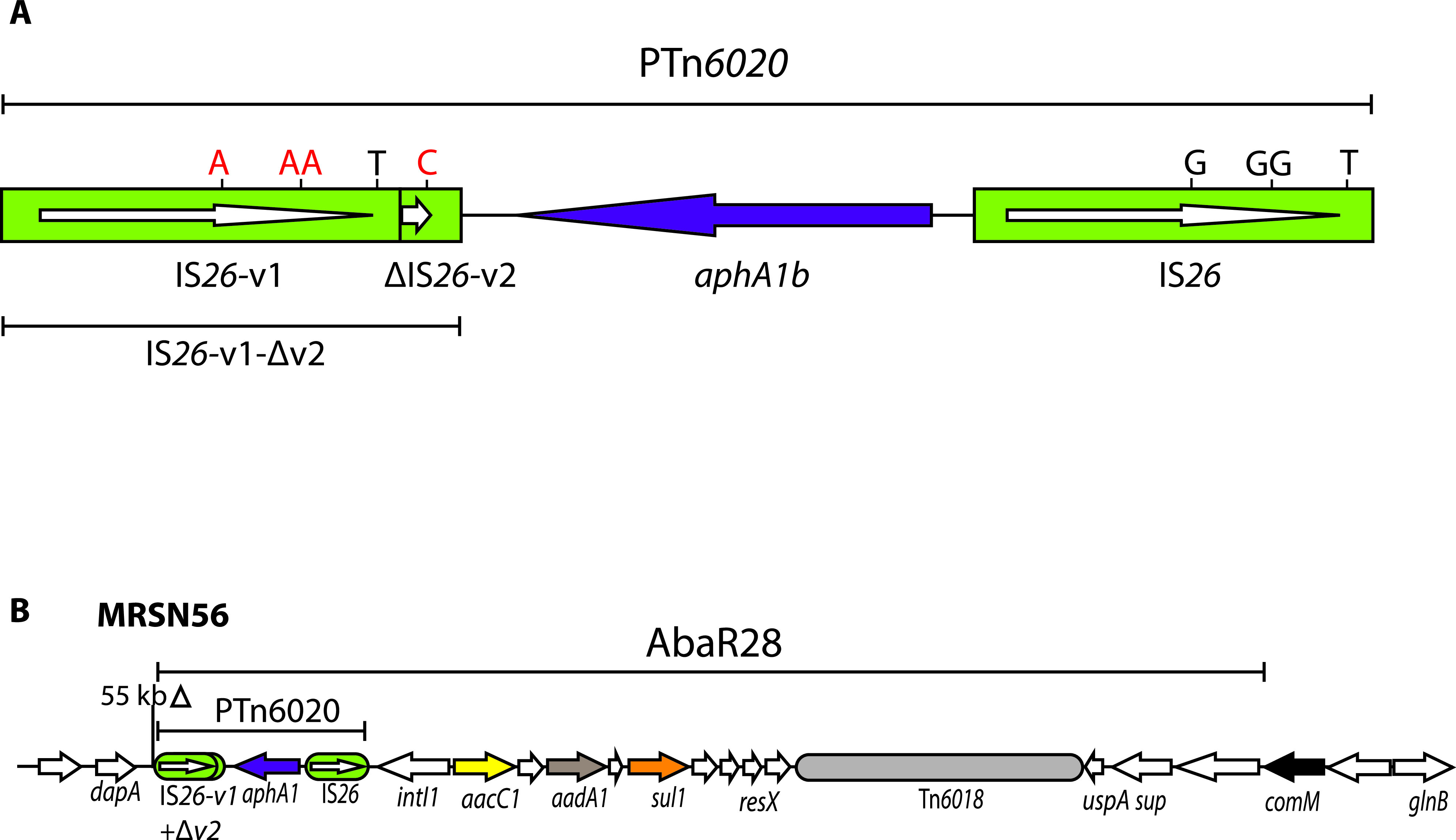
(A and B) Schematics showing the structure of PTn*6020* (A) and AbaR28 (B) in MRSN56. IS*26*s are denoted by green boxes, with internal arrows showing the extent and orientation of the *tnp26* transposase gene. The *aphA1b* kanamycin and neomycin resistance gene is denoted by a purple arrow, and other resistance genes are colored. Structures of known origin are labeled. In A, letters above the vertical lines denote the nucleotides that differentiate IS*26* from IS*26*-v1 and IS*26*-v2, with wild-type bases in black letters and changed bases in red. Drawn to scale from GenBank accession number CP080452.

MRSN56 is an extensively resistant GC1/CC1 Acinetobacter baumannii isolate recovered in 2010 from the wounds of a 20-year-old soldier injured in Afghanistan and returned to the United States for treatment (4). Treatment with tobramycin, one of the few remaining options, led to tobramycin resistance arising from substantial amplification of the *aphA1* gene (4). The *aphA1* gene normally confers resistance to kanamycin and neomycin but not tobramycin, as AphA1 cannot modify tobramycin. However, it can sequester tobramycin via stoichiometric binding (7), and expression of sufficiently high levels of the enzyme can lead to the observed tobramycin resistance. The MRSN56 chromosome (4, 8) (GenBank accession number CP080452) includes a single copy of *aphA1* carried by the pseudocompound transposon PTn*6020* (formerly Tn6*020*) (9) and located in the AbaR28 resistance island (Fig. 1), but substantial amplification of *aphA1* was detected using a variety of methods in the MRSN56 derivatives MRSN57 and MRSN58, isolated independently after tobramycin therapy began (4). From the short-read data available at the time, it was deduced that the segments amplified included PTn*6020* together with additional adjacent DNA, and, in the case of MRSN58, the amplified segment was not located near the original PTn*6020* copy. These features are not consistent with an unequal crossing over model for gene amplification involving initially the duplicated IS*26*, which would produce tandem duplications of just the PTn*6020* adjacent to the original copy.

**FIG 2:**
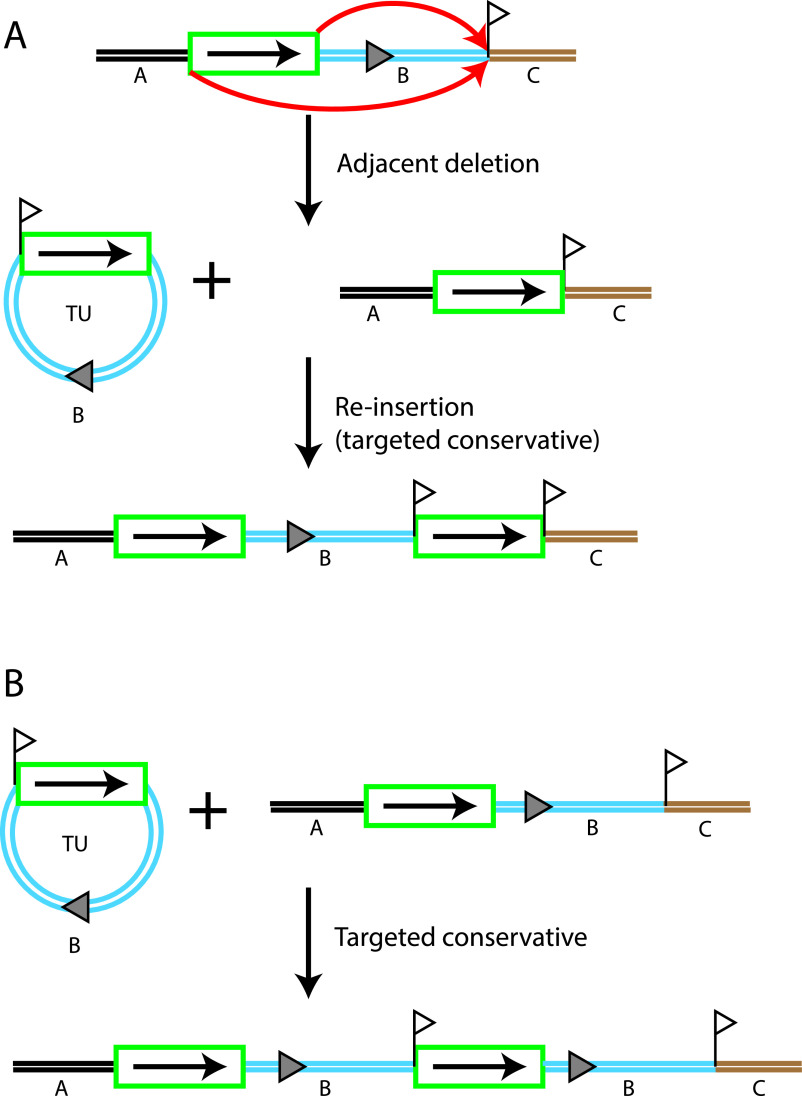
TU formation and two routes to TU reincorporation. (A and B) Adjacent deletion forms a TU followed by targeted conservative cointegration into the deletion product (A) and reincorporation of the TU into the original structure by targeted conservative cointegration (B). IS*26* is denoted by a green box, and different DNA segments are labeled and colored. Target site duplications or the 8 bp belonging to a particular site are denoted by a raised flag. A gray triangle indicates the orientation of segment B.

IS*26* is widely spread in antibiotic-resistant Gram-negative pathogens, including A. baumannii, and plays a key role in the recruitment and further dissemination of resistance genes. Its importance is a consequence of the dual mechanisms used to move IS*26*. IS*26* only forms cointegrates rather than undergoing simple transposition (10) but uses two distinct mechanisms (11–13). In the better-known reaction, cointegrate formation is achieved via a “copy-in” route (formerly known as “replicative transposition”), which involves the reaction between IS*26* and a randomly selected target sequence, followed by a replication step that duplicates the IS. This process also generates an 8-bp duplication of the target site (13–15). If the target site is on the same DNA molecule as the IS, a deletion of adjacent DNA can arise (13, 14, 16, 17). As shown in Fig. 2A, this produces a circular nonreplicating molecule that includes a single copy of the IS together with adjacent DNA known as a translocatable unit (TU) (13). A single copy of the IS is also left behind at the original location, and the target site duplication (TSD) is distributed between the two molecules. The TU can be incorporated at a new site using the copy-in route, forming a novel pseudocompound transposon bounded by directly oriented copies of IS*26* and flanked by a duplication of the target site (14, 18).

However, it is now known that IS*26* also uses a more efficient targeted conservative cointegration route that involves the reaction between IS*26* copies in different DNA molecules, and this route does not generate any additional DNA (13). This process involves a Tnp26-catalyzed single-strand transfer at one end of each of two ISs (either pair of like ends) if the ISs are in different DNA molecules (11). This is followed by branch migration (12). How this intermediate is finally resolved is not currently known, but replication initiated at a free 3′ end could occur. This route is 100- to 1,000-fold more efficient than the copy-in route (11, 13) and also at least 100-fold more efficient than homologous recombination within the two IS*26*s, which could produce the same product (19). Using this route, a TU (formed as described above) can be reinstated next to the IS*26* in the second deletion product, resulting in a duplication of only the IS*26* (Fig. 2A). However, if it is incorporated next to a copy in an intact original configuration present in the same cell, the TU would be duplicated (Fig. 2B). Duplication via this route was recently reported in the case of a carbapenem resistance gene in the A. baumannii plasmid pACICU1 (20).

If sufficient sequence information is available, the different characteristics of copy-in and targeted conservative cointegration reactions can enable distinctions to be drawn between events that occur by one mechanism or the other. In addition, PTn*6020* consists of a short DNA segment containing the *aphA1* gene surrounded by IS*26* variants, IS*26*-v1, and a partial copy of IS*26-*v2 (here IS*26*-v1-Δv2) on the left and IS*26* on the right (Fig. 1A) (9). The differences that distinguish IS*26*-v1 and IS*26*-v2 from IS*26* have the potential to assist in distinguishing events mediated by the IS*26-*v1 on the left from those involving the IS*26* on the right.

MRSN56 is an ideal candidate for examination of the amplification mechanism as it carries a single copy of PTn*6020* in the AbaR28 resistance island (Fig. 1B), and no additional IS*26* copies were found in the complete genome, which we determined recently (8). The ability to examine the detail of the chromosomal alterations leading to the emergence of tobramycin resistance is limited using just short-read data. Consequently, here, the complete genomes of the tobramycin-resistant MRSN57 and MRSN58, which arose from MRSN56 during therapy, were determined. Changes in the chromosome relative to the complete genome of MRSN56 were examined in light of the known IS*26*-mediated reactions. Complete or draft genomes of tobramycin-resistant derivatives of MRSN56 recovered previously and isolated here in the laboratory by exposure of MRSN56 to increasing concentrations of tobramycin were also examined.

## RESULTS

### Structure of the amplified region in MRSN58.

**FIG 3:**
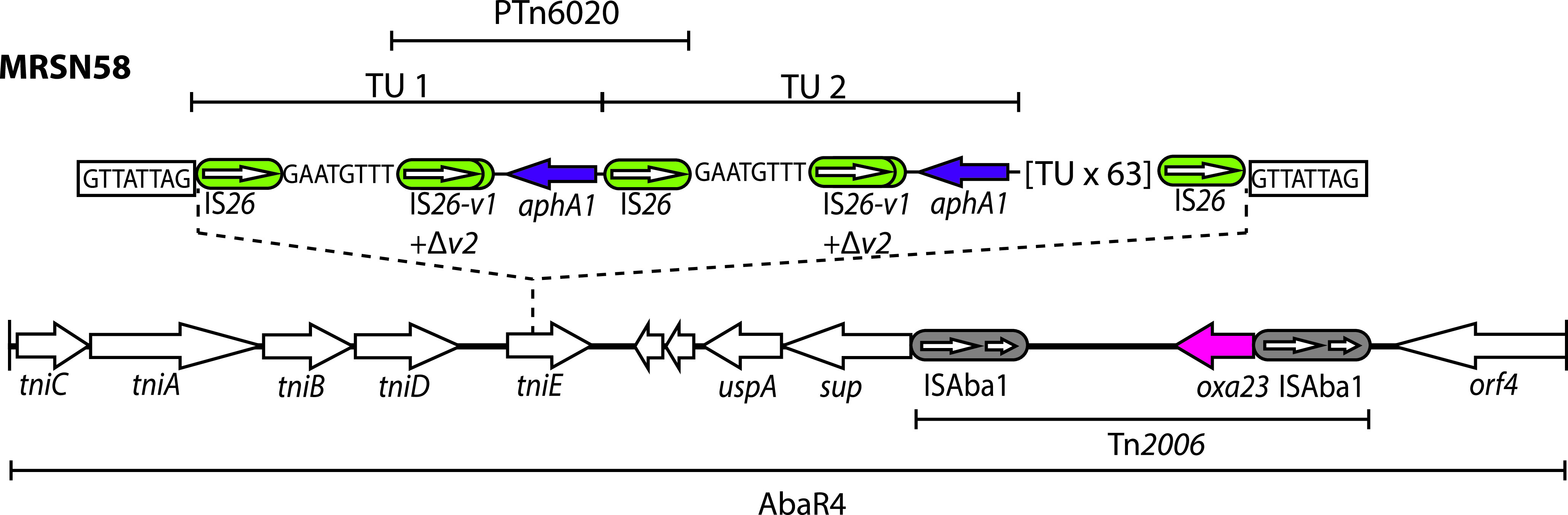
Structure of AbaR4 in MRSN58 with tandem amplification of PTn*6020*. IS*26*s are shown as green rounded boxes, and the orientation and extent of genes are indicated by horizontal arrows. Structures of known origin are labeled. The nucleotide sequence of the target site duplication generated by insertion of PTn*6020* into AbaR4 in MRSN58 is shown in a box. The eight nucleotides included in the translocatable unit (TU) generated from AbaR28 are shown without a box. Drawn to scale from GenBank accession numbers CP080452 and CP090607.

Previously, the amplified *aphA1*-containing segment in MRSN58 was shown to be located away from the original copy of PTn*6020* in a structure identified here as AbaR4 that is found 680 kbp from AbaR28. The insertion is located in the *tniE* gene of AbaR4 (Fig. 3). However, the structure of this additional segment shown previously (Fig. 2b in McGann et al. [4]) lacked an IS*26* adjacent to the TSD at the left end that would be expected for a copy-in mechanism. In addition, it has been proposed that the structure represents a misinterpretation of the sequence data (21).

Here, the complete genome of MRSN58 was determined and was examined in light of this previous report. An IS*26* was found at both ends of the amplified region immediately abutting the TSD (Fig. 3). The insertion consists of tandem copies of a 3,077-bp TU made up of PTn*6020* and an additional 8 bp that are found to the left of PTn*6020* in AbaR28 (as shown in Fig. 1A). Up to 6 copies of this TU were found in the longest reads, and examination of all the long reads used in the assembly yielded no cases where the 8-bp segment is missing from between the IS*26*-flanked *aphA1*-containing segments. Hence, the internal structure is as originally proposed. The number of TU copies was previously estimated to be 65 using multiple methods (4), and 65 copies were therefore included in the final chromosome assembly (CP090607).

### Formation of the amplified region in MRSN58.

**FIG 4:**
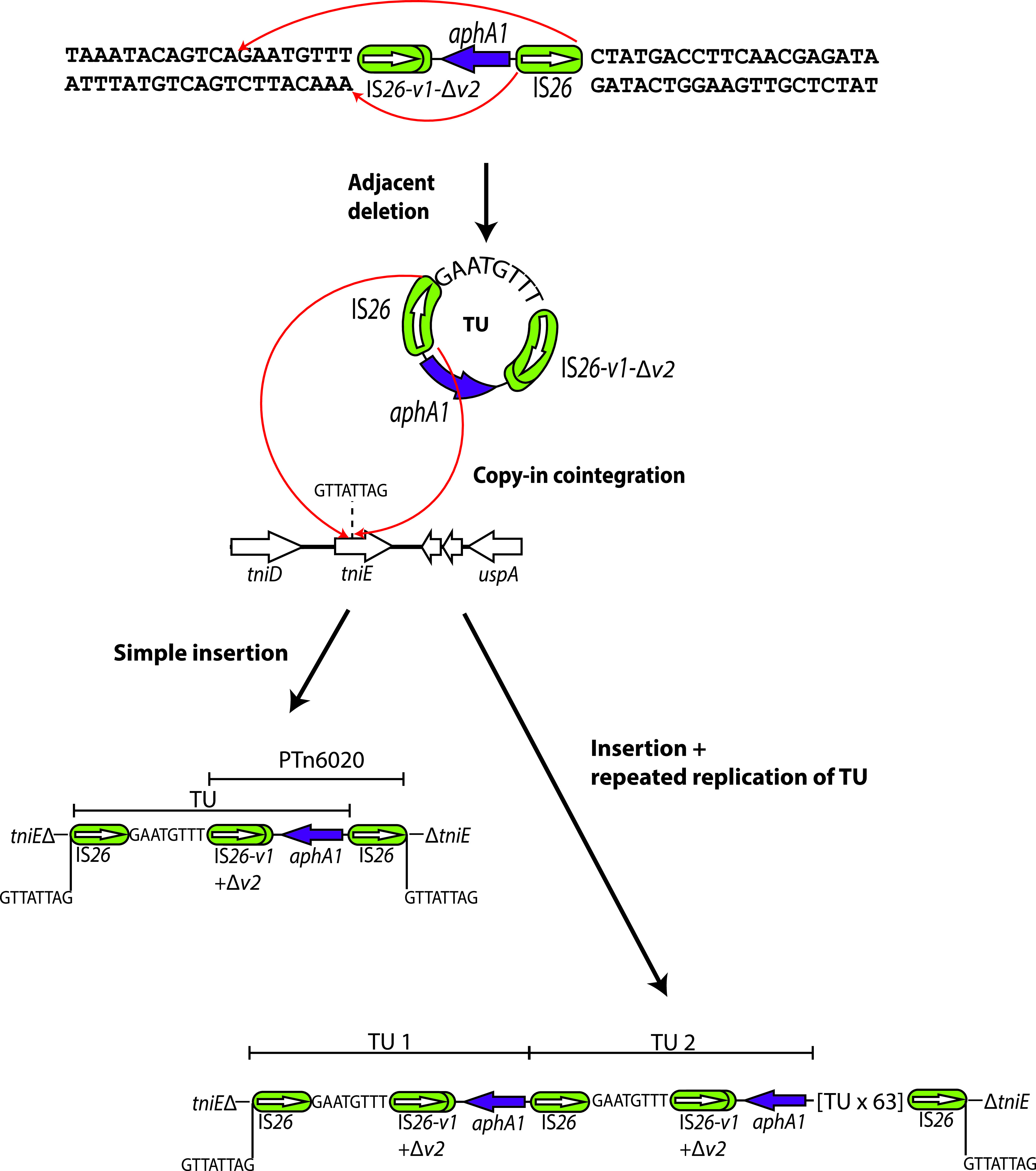
Generation of a translocatable unit (TU) and insertion into AbaR4 in MRSN58. IS*26*s are shown as green rounded boxes, and the orientation and extent of genes are indicated by horizontal arrows. Structures of known origin are labeled. The target site duplication is denoted by a vertical line and letters. Red arrows indicate the site of action of IS*26* to generate a TU via adjacent deletion (top) and to incorporate a TU into a new location by copy-in cointegration. Drawn to scale from GenBank accession numbers CP080452 and CP090607.

The route to formation and relocation of the amplified segment is shown in Fig. 4. The TU would have formed via the action of the IS*26* on the right side of PTn*6020* to form a circular TU molecule. Here, the AbaR28 resistance island was found to be intact in MRSN58, indicating that the copy of the chromosome retained was not the one from which the TU was formed. The IS*26* in the TU has then inserted the TU into the *tniE* gene of AbaR4 using the copy-in mode. Simple insertion would yield only a single copy of the TU and an additional copy of IS*26* flanked by the TSD. As the initial unequal crossing over event could involve any pair of IS*26*, this route would likely not yield a homogeneous tandem duplication of the TU observed. Hence, it seems more plausible that the replication step required to complete one of the copy-in processes (either TU formation or TU reinsertion) has continued around the TU circle multiple times (rolling circle replication). This could potentially have occurred during the formation of the TU or during its insertion at the new location and would directly link amplification to the Tnp26-mediated formation of the TU or its integration at a new site (as shown in Fig. 4).

### Structure and formation of the amplified region in MRSN57.

**FIG 5:**
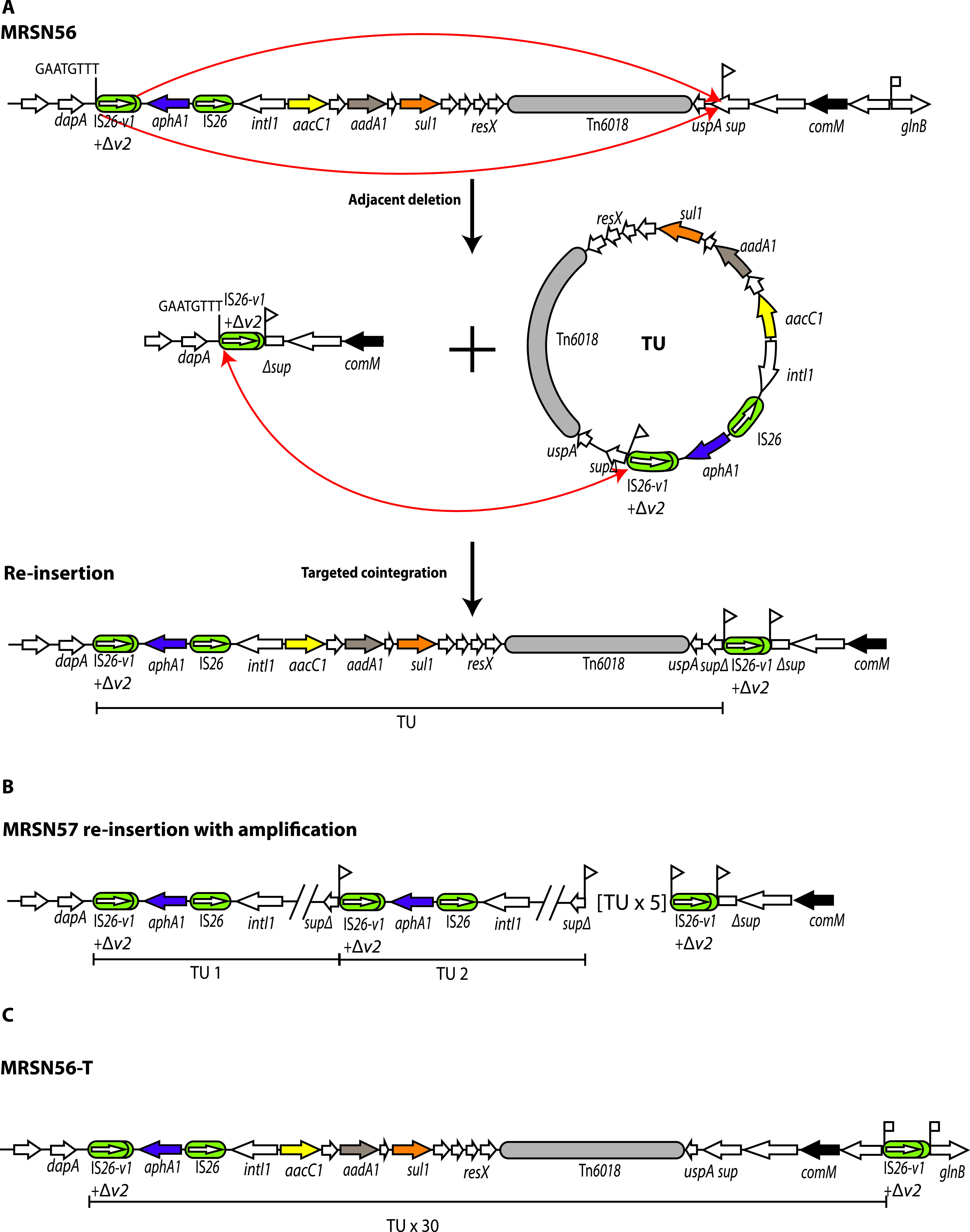
(A to C) Generation of a TU via adjacent deletion in AbaR28 followed by simple reinsertion (A) and tandem amplification in MRSN57 (B) and MRSN56-T (C). IS*26*s are shown as green rounded boxes, and the orientation and extent of genes are indicated by horizontal arrows. Structures of known origin are labeled. Target site duplications are denoted by vertical flags. Red arrows indicate the site of action of IS*26* to generate a TU via adjacent deletion and to incorporate a TU by targeted conservative cointegration adjacent to an existing IS*26*. The number of TUs in the tandem array are denoted. Drawn to scale from GenBank accession numbers CP080452 and CP091172.

The second clinical isolate MRSN57 was previously found to include several copies of PTn*6020* as part of a large segment of AbaR28 located adjacent to its original position (4). However, using only short-read data, the exact configuration could not be deduced readily. From the complete genome determined here, it was possible to provide a more detailed picture of the amplified region. The repeated unit was 15,207 bp in length, and the longest reads included 2 copies of this repeat in tandem. As the copy number of 7 reported previously was consistent with the copy number determined here (estimated using the read depth of *aphA1* relative to that of averaged multilocus sequence typing [MLST] markers), 7 copies were included in the final assembly (CP091172). It was also clear that the copy of IS*26* at the right end of the amplified region is IS*26*-v1-Δv2, and it is surrounded by an 8-bp TSD (Fig. 5A).

The presence of this TSD allowed the route to be deduced. As shown in Fig. 5B, in this case, one of the routes shown in Fig. 2 was used. The 15,207-bp TU was formed via adjacent deletion initiated by the IS*26*-v1-Δv2 forming a TSD that is distributed between the TU and the deletion remnant. In order to generate the final configuration observed, these two molecules must have come together again in a targeted conservative reaction occurring at one end of the IS*26*-v1-Δv2 or possibly via homologous recombination occurring within the IS*26*-v1Δv2. However, a simple reinsertion step using either the targeted conservative route or homologous recombination would only recreate the original configuration of AbaR28 with an additional IS*26*-v1-Δv2 in the *sup* gene that is surrounded by a TSD. However, if IS*26* was involved, it can be envisaged that resolution of one of the initial branched products formed by Tnp26 action has occurred only after repeated rounds of replication through the TU, and this would lead to the amplified structure observed. In contrast, if the simple insertion step occurred via homologous recombination, an initial round of unequal crossing over involving the two IS*26*-v1-Δv2 elements would then be needed to generate a duplication of the TU. Only then could further amplification occur via further rounds of unequal crossing over involving the larger duplicated TU segment.

### Structure and formation of the amplified region in MRSN56-T.

Only short-read data were available for MRSN56-T, a tobramycin-resistant derivative of MRSN56, which arose during the course of growth in the laboratory on an increased concentration of tobramycin (4). However, examination of the relevant boundaries of IS*26* with adjacent DNA revealed that it was possible to deduce that IS*26*-v1-Δv2 is at the right end of the amplified region and is surrounded by an 8-bp TSD (Fig. 5C). Hence, the pathway involved in the formation of the amplified region of MRSN56-T was similar to that shown for MRSN57. Again, the 19,827-bp TU was formed by IS*26*-v1-Δv2 and reincorporated at the same site, creating an additional copy of IS*26*-v1-Δv2 surrounded by a TSD in the chromosome beyond the right end of AbaR28 (Fig. 5C). Amplification, which yielded approximately 30 copies of the TU, would require repeated replication through the TU circle in the intermediate formed by Tnp26 during its formation or reinsertion.

### Experimental amplification.

**FIG 6:**
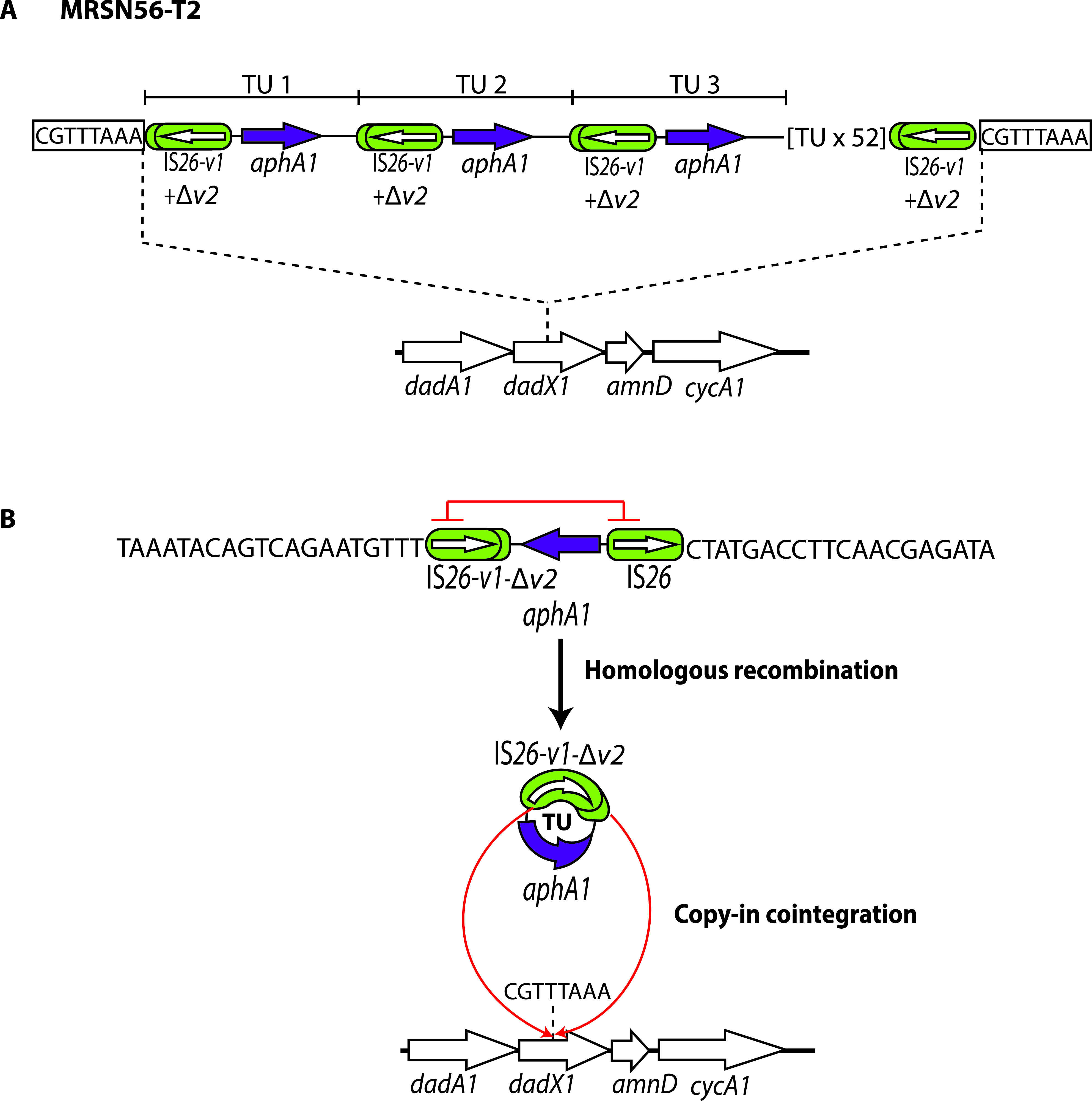
(A and B) Experimental induction of a tandem repeat in MRSN56-T2 following 10 days of growth under increasing concentrations of tobramycin (A) and generation of a TU from MRSN56 via homologous recombination, followed by copy-in cointegration into a new location (B). IS*26*s are shown as green rounded boxes, and the orientation and extent of genes are indicated by horizontal arrows. Structures of known origin are labeled. Target-site duplications are denoted boxed letters. Red bars indicate the region of each IS*26* in which homologous recombination would have occurred, and red arrows indicate the site of action of IS*26* to incorporate a TU by copy-in cointegration at the new location in MRSN56-T2. The number of TUs in the tandem array are denoted. Drawn to scale from GenBank accession numbers CP080452 and CP090606.

MRSN56 was again grown overnight on medium containing increasing concentrations of tobramycin, and DNA prepared from samples taken after 5 to 9 days of exposure to tobramycin (concentrations increasing from 1 to 16 μg/mL) was sequenced (MinION data). At every time point, the amplified segment was made up of a 2,249-bp TU containing only IS*26*-v1-Δv2 and the central segment of PTn*6020*. Copies of this TU in tandem were found at a new location in the *dadX1* gene (locus tag FMBFHNBO_00654 in CP080452), which is between KL1 and AbaR28 (Fig. 6A). This tobramycin-resistant variant was designated MRSN56-T2. The longest reads included 11 copies of this TU, based on the relative read depth for *aphA1*, and the TU was already amplified 19 times when first examined at day 5 (4 μg/mL tobramycin). The number of copies estimated increased when the tobramycin concentration was increased to 24 on days 6 and 7 (tobramycin concentration of 8 μg/mL), 78 on day 8, and 56 on day 9 (both 16 μg/mL). The higher copy number on days 8 and 9 could have occurred via unequal crossing over.

To examine the mechanism by which MRSN56-T2 arose, the complete sequence of MRSN56-T2 in the day 9 sample was determined, and AbaR28 was found to be intact. In this case, the 2,249-bp TU appears to have arisen via homologous recombination between the IS*26*-v1-Δv2 and IS*26* flanking the *aphA1* gene and was incorporated at the new location via the copy-in route, creating an 8-bp target-site duplication (Fig. 6B). Again, significant amplification likely occurred in the course of resolution of the intermediate formed in the initial Tnp26-catalyzed TU incorporation step.

## DISCUSSION

MRSN57 and MRSN58 are each clear examples of heteroresistance that arose via extensive amplification of segments that include the *aphA1* gene, leading to tobramycin resistance. The detail embedded in the complete genome sequences determined here combined with the availability of a complete genome for the susceptible parent isolate MRSN56 were key to allowing the amplification mechanism to be deduced using insights derived from recent detailed studies of the mechanisms used by IS*26.* The most often proposed route to amplification involves unequal crossing over between duplicate copies of a sequence in the chromosome or in a plasmid (2, 22), but no evidence was obtained for this route. In the case of PTn*6020*, this would first involve recombination between the copies of IS*26* at the outer boundaries. Recombination would lead to an additional copy of the central portion of this transposon together with a single IS*26* copy (IS*26*-v1-Δv2 or IS*26*) located adjacent to the original copy in AbaR28 in one of the recombining chromosomes. No TSD would be generated. However, none of the four independently derived MRSN56 derivatives had this structure. In addition, a target-site duplication was generated in each of the four independent cases of amplification examined here, and this led us to invoke an alternate IS*26*-dependent mechanism for the observed extensive amplification that relied on the action of IS*26*.

For MRSN58 and MRSN56-T2, the TSD and IS*26* duplication at the boundaries of the amplified region can be explained by the fact that the amplified segment is in a new location, and a TU, generated via copy-in adjacent deletion for MRSN58 (Fig. 4) or via homologous recombination for MRSN56-T2 (Fig. 6), must have been incorporated there using the copy-in route, which generates the extra IS and the TSD. However, in the case of MRSN57 and MRSN56-T, where the larger amplified region is associated with AbaR28, the appearance of an additional IS*26* flanked by a TSD cannot be explained by any homologous recombination mechanism. Nor can simple transposition of an IS*26* explain this observation, as a recent study has established that simple transposition does not occur (10). Instead, whether using the copy-in or the targeted conservative mode, the action of Tnp26, the transposase encoded by IS*26*, always generates a cointegrate, either between two replicons (plasmid and chromosome or two plasmids) or between a circular TU and a replicon. Therefore, to generate the structures found in MRSN57 and MRSN56-T, IS*26* has first formed a TU and an adjacent deletion, which occurs via a copy-in route, and hence duplicates the IS and generates a TSD (Fig. 2). These two molecules each containing one copy of the TSD have then been brought back together via the targeted conservative route, as shown in Fig. 2 and 5. The extensive amplification observed in these cases can best be explained by invoking multiple rounds of replication through a TU in the initial intermediate formed by Tnp26 at the TU excision or TU reincorporation step. Although the reuniting of the products of the adjacent deletion event could potentially occur via homologous recombination, this route would not facilitate subsequent coupled amplification.

IS*26* has often been shown or deduced to be involved in the amplification of a DNA segment, leading to elevated levels of resistance to one or more specific antibiotics, although generally the number of copies is modest (3, 23–27). However, two further cases of extensive amplification have been reported (5, 6). A highly amplified segment (170 to 190 copies) consisting of the *bla*_TEM-1_ gene and a single IS*26* was found in the chromosome of a clinical Escherichia coli isolate that was resistant to piperacillin-tazobactam. The amplified region was bounded by IS*26* copies and flanked by a TSD (6). The structure is similar to that of the MRSN56-T2 derivative shown in Fig. 6 and may have arisen in a similar way. However, this could not be investigated here because the sequence of the initial susceptible isolate is not available. The structure was relatively stable, as the copy number only reduced to 70 or 36 copies after 150 generations without selection. The second case of extensive amplification also involved the *bla*_TEM-1_ gene, with up to 113 copies found in a resistant E. coli isolate (5). However, the structure of the amplified segment was not clearly described, and whether a TSD flanked the amplified region was not reported. We therefore examined the available genome sequence and long reads (PRJNA431448) and found that the chromosome was open at a point within the amplified segment, and the amplified segment was in an additional contig. This is likely due to assembly difficulties arising from the fact that the TU is long. However, from the long-read data, it was clear that the amplified region was in the chromosome and was made up of tandemly repeated copies of an 11.6-kbp segment (a TU) containing the *dfrA17*, *aadA5*, *sul1*, and *bla*_TEM-1_ genes and a copy of IS*26.* The amplified region was bounded by directly oriented copies of IS*26*, and a TSD was found flanking the region. In both of these cases, if the original susceptible isolate (for which no sequence is available) included a single copy of this TU in the same location, amplification could have occurred by unequal crossing over or alternatively via homologous recombination leading to excision of the TU as a circle followed by its incorporation into a sister chromosome adjacent to one of the IS*26*s, again via homologous recombination. However, reincorporation of the TU could have been catalyzed by Tnp26 using the conservative mechanism and thus could have used the novel amplification mechanism proposed here.

A further case of amplification of the *bla*_TEM_ gene in a piperacillin-tazobactam-resistant clinical E. coli isolate recovered from a patient that had been treated with this antibiotic/inhibitor combination was reported recently (28). Its sequence was compared to that of a closely related susceptible isolate recovered previously from the same patient. In this case, the large size of the TU (10.9 kbp) again led to assembly issues for the chromosome with the amplified array, which was open at this position but carried a remnant of IS*26* on only one end. However, the authors concluded that the additional copies were in this location. The sequence of the putative parent was also not complete. Here, both chromosomes were reassembled, and the assembly problems traced to low average read length for the long-read data (6,568 bp). Although our assembly confirmed that the amplified segment was at the location of the original IS*26*-bounded segment, and there were no further copies of IS*26* elsewhere in the chromosome, the availability of the sequence of the susceptible precursor did not allow the mechanism to be clearly defined for the reasons described above.

The IS*26*-dependent route to extensive amplification proposed here would continue to occur in the absence of homologous recombination, which is needed for unequal crossing over. This has been observed in an early case of amplification of an IS*26*-associated segment (26, 27), but, as the sequence revealing the precise structure and location of the amplified region is not available, we could not examine how IS*26* was involved. Further work will be needed to determine if amplification does occur in a recombination-deficient background. Finally, it is likely that the route to extensive amplification proposed here will also be available to members of the IS*26* family (as defined recently [29]). In particular, IS*1216* found in Gram-positive pathogens and IS*1006*, IS*1008*, and relatives found in Acinetobacter species have been shown to perform the same reactions as IS*26* (12, 30) and these ISs are found associated with antibiotic resistance genes.

## MATERIALS AND METHODS

### Bacterial strains.

Acinetobacter baumannii strains MRSN56, MRSN57, MRSN58, and MRSN56-T were described previously (4). The complete genome of MRSN56 was previously determined by combining long-read (Oxford Nanopore) and short-read (Illumina MiSeq) data in a hybrid assembly (8).

### Experimental amplification.

Induction of tobramycin resistance was achieved by growing MRSN56 on increasing concentrations of tobramycin over a 10-day period. MRSN56 was first streaked onto a nutrient agar plate without antibiotic and grown for 24 h at 37°C. A single colony was then streaked on a nutrient agar plate containing 1 μg/mL tobramycin (day 1). Thereafter, every 24 h, a colony was picked and restreaked onto a nutrient agar plate containing tobramycin at the following concentrations: 1 μg/mL (days 2 to 3), 2 μg/mL (day 4), 4 μg/mL (days 5 to 6), 8 μg/mL (days 7 to 8), and 16 μg/mL (days 9 to 10). On days 6 to 10, a single colony was picked for further analysis.

### Genome sequencing.

Draft assemblies of MRSN57, MRSN58, and MRSN56-T were previously obtained from short-read Illumina MiSeq data (4). Here, MRSN57 and MRSN58 DNA prepared previously was resequenced on an Oxford Nanopore MinION according to manufacturer’s instructions to obtain long-read data. DNA was extracted from single tobramycin-resistant colonies of MRSN56 sampled after 5 to 9 days of growth on tobramycin using a TruSeq DNA kit (Illumina), according to the manufacturers’ instructions, and also sequenced on an Oxford Nanopore MinION. DNA from the day 10 isolate was further sequenced on an Illumina MiSeq platform according to manufacturer’s instructions. This derivative was designated MRSN56-T2.

### Genome assembly.

Due to excessive read depth (~1,000×), the MinION reads were first filtered using Fitlong version 0.2.1 (https://github.com/rrwick/Filtlong) to remove reads larger than 1,000 bp, and the output was reduced to 500 Mbp of long reads. The MRSN57 and MRSN58 long reads were combined with the previously obtained short reads using Unicycler version 0.4.0 with default settings (31, 32) to produce a hybrid assembly. The original MRSN56-T draft assembly had been trimmed to remove IS ends, which prevented proper analysis of the tandem unit, so the available short reads were reassembled using Unicycler with default short-read assembly parameters to produce a new draft assembly. A hybrid assembly for MRSN56-T2 at day 9 was produced by combining long and short reads determined here using Unicycler with default settings (31, 32), while for days 5 to 8, long reads were assembled using Unicycler with default long-read-only settings. All of these assemblies were modified to include the appropriate number of TUs determined from the read coverage relative to that of chromosomal MLST markers. The chromosomes were annotated in line with and as described for MRSN56 (8).

### Analysis of tandem repeat regions.

Tandemly repeated regions in new locations were found by searching for all IS*26* locations using a standard IS*26* sequence and comparing to the complete MRNS56 sequence (GenBank accession number CP080452) as a reference scaffold. The structure of the individual repeat units was determined by examining the hybrid or long-read assemblies and comparing them to the PTn*6020* and AbaR28 reference sequences. As the number of tandem repeats was too great to be resolved in a hybrid or long-read assembly, a read-based approach was used to determine the number of copies of the tandem repeat by examining the number of copies of the *aphA1* kanamycin and neomycin resistance gene relative to the chromosomal Oxford MLST markers as follows. Seqtk (https://github.com/lh3/seqtk) was used to perform a fastq-dump to convert the Filtlong-processed 500 Mbp of long-read data from each sample into a fasta file, which was then compiled into a custom stand-alone BLAST+ database. The database was queried with the *aphA1* gene and the MLST markers. The total number of hits for each query was compiled into a .csv file, and the copy number of the tandem repeats was determined by dividing the total number of *aphA1* hits by the average number of hits for the Oxford MLST markers. The read depth of *aphA1* relative to the rest of the chromosome was also visually confirmed by using a Minimap2 (https://github.com/lh3/minimap2) and Samtools (https://github.com/samtools) pipeline to align and index the reads to the MRSN56 reference sequence before visualizing in the Integrative Genomics Viewer (version 2.11.3).

To find diagnostic single-nucleotide differences in the IS that allow for the movement of individual ISs to be tracked, copies of IS*26* in each final assembly were examined manually using sequences of IS*26* and variants (33, 34). The surrounding sequence was also examined manually to identify target site duplications (TSDs).

### Data availability.

The sequences of the chromosomes of MRSN57, MRSN58, and MRSN56-T2 have been deposited in GenBank (BioProject PRJNA742487) under accession numbers CP091172, CP090607, and CP090606, respectively. Reads for all samples and the draft assembly of MRSN56-T are available under the BioSample and SRA accession numbers listed in Table 1.

**TABLE 1:** GenBank accession numbers, BioSample, and SRA entries for BioProject PRJNA742487

Sample	BioSample	MiSeq	MinION	Chromosome accession
MRSN56	SAMN19955388	SRR14998418	SRR14998417	CP080452 ^*a*^
MRSN57	SAMN23416895	SRR17023755	SRR17023754	CP091172
MRSN58	SAMN23416908	SRR17023784	SRR17023783	CP090607
MRSN56-T	SAMN19966358	SRR15084659	NA	NA
MRSN56-T2 day 5	SAMN20203777	NA	SRR15128354	NA
MRSN56-T2 day 6	SAMN20203779	NA	SRR15128356	NA
MRSN56-T2 day 7	SAMN20203785	NA	SRR15128362	NA
MRSN56-T2 day 8	SAMN20203933	NA	SRR15128546	NA
MRSN56-T2 day 9	SAMN20178847	SRR15115349	SRR15115348	CP090606

a Plasmids found in MRSN56 are listed under CP080453 to CP080456; NA, not available.
